# Biomass-Derived Carbon Dots: Preparation, Properties, and Applications

**DOI:** 10.3390/nano15161279

**Published:** 2025-08-19

**Authors:** Qinfeng Liu, Huan Chen, Ruiyu Mi, Xin Min, Minghao Fang, Xiaowen Wu, Zhaohui Huang, Yangai Liu

**Affiliations:** Engineering Research Center of Ministry of Education for Geological Carbon Storage and Low Carbon Utilization of Resources, Beijing Key Laboratory of Materials Utilization of Nonmetallic Minerals and Solid Wastes, National Laboratory of Mineral Materials, School of Materials Science and Technology, China University of Geosciences, Beijing 100083, China

**Keywords:** biomass-derived carbon dots, synthesis, properties, applications

## Abstract

With the intensification of the global energy crisis, green, low-carbon, and environmentally friendly biomass materials have become the focus of research. Among them, biomass-derived carbon dots (B-CDs), a novel class of sustainable zero-dimensional carbon nanomaterials, attract significant interest due to their environmental friendliness, low toxicity, and unique optical properties. Research findings indicate that B-CDs, utilizing biomass materials as carbon sources, demonstrate significant potential in numerous application fields through structural design and photo-functionalization. However, the underlying mechanisms and formation processes of B-CDs remain inadequately elucidated, and systematic summarization still requires further refinement. Therefore, this review systematically summarizes the synthesis methods, precursor structures, formation mechanisms, luminescent properties, and prevailing applications of B-CDs, with a particular emphasis on recent advances in their use for sensing, anti-counterfeiting, bioimaging, and optronics. In addition, the challenges encountered in performance-oriented controllable preparation and large-scale production were also clarified. This comprehensive review provides a theoretical foundation for further research and multidisciplinary applications of B-CDs, thereby contributing to promoting large-scale commercialization and industrial implementation.

## 1. Introduction

### 1.1. Carbon Dots

**Figure 1:**
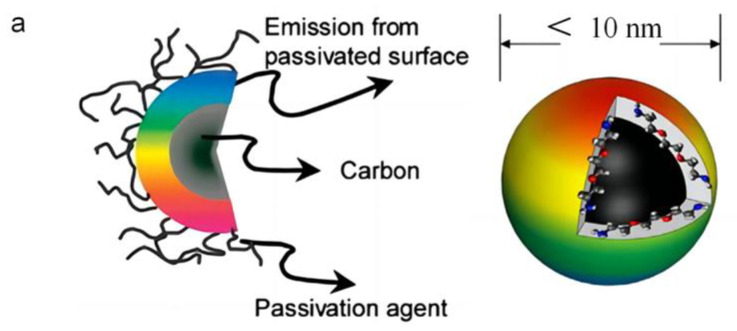

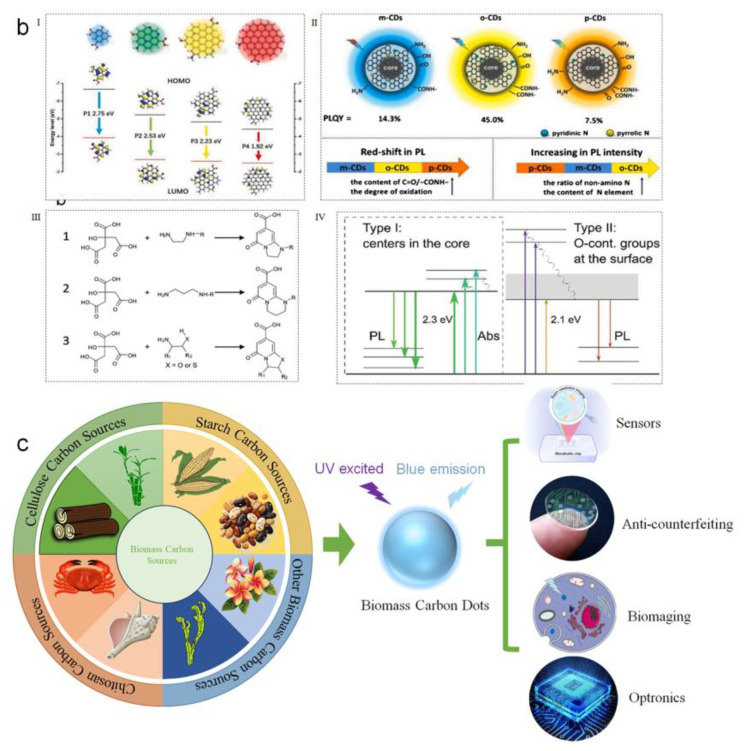
(**a**) Structural configuration of carbon dots [11]. (**b**) Luminescence mechanism diagrams of carbon dots: (**I**) Mechanism diagram of photo-tunable fluorescence via quantum confinement regulation in CDs [12]. (**II**) Schematic illustration of multicolor CD synthesis through surface functional group tuning [12]. (**III**) Molecular fluorophore states in citric acid-based carbon polymer dots (CPDs) derived from varied nitrogen precursors [9]. (**IV**) Oxygen-containing surface groups as CD emission origin (left). Post-UV irradiation: carbon core-dominated optical centers (right) [10]. (**c**) Carbon precursors and implementation scenarios for CDs.

The accelerated pace of industrialization and urbanization has led to a rise in escalating energy consumption and environmental pollution. Consequently, achieving carbon peak and carbon neutrality has become an urgent priority for the international community [1]. In recent years, environmental friendly carbon-based nanomaterials have emerged as viable options for many optical applications because of their remarkable thermal conductivity, excellent electrical properties, high mechanical strength, and varied optical features [2]. Carbon dots (CDs), among the representative carbon-based nanomaterials, exhibit exceptional photoluminescence properties [3]. Fluorescent carbon nanoparticles were initially described in 2004 when Xu et al. inadvertently found them during the purification of single-walled carbon nanotubes using an arc-discharge process [4]. In 2006, Sun et al. attained high-quantum-yield carbon dots by chemical passivation methods, officially introducing the name “carbon dots” [5]. CDs are characterized as surface-passivated, zero-dimensional carbon nanostructures, often possessing diameters under 10 nm, displaying a core–shell architecture (Figure 1a) [6]. This structure consists of an sp^2^/sp^3^-hybridized carbon core enveloped by an oxygen-functionalized shell passivated with molecular moieties such as carboxyl and hydroxyl groups. With advances in synthesis strategies for CDs, the elucidation of their photoluminescence mechanisms has become increasingly critical.

CDs primarily exhibit four photoluminescence mechanisms. (i) Quantum Confinement Effect: The quantum confinement effect occurs when the size of CDs approaches or falls below their exciton Bohr radius, causing quasi-continuous energy levels near the Fermi energy to split into discrete states. This spatial confinement of charge carriers increases the bandgap and induces a blue shift in emission wavelengths (Inset (I) in Figure 1b) [7]. (ii) Surface State Emission: Surface state emission arises when surface functional groups on CDs trap electrons or holes to form surface states, which act as centers for electronic transitions and participate in luminescence. As shown in inset (II) of Figure 1b, tuning these surface groups enables the synthesis of multicolor CDs [8]. (iii) Molecular State Fluorescence: Molecular state fluorescence is predominantly observed in CDs synthesized via bottom-up approaches (Inset (III) in Figure 1b). During carbonization, intrinsic fluorophores or newly formed emissive moieties embedded in these CDs remain undecomposed, retaining independent electronic transition properties. These moieties function as fluorescent centers analogous to organic fluorophores [9]. (iv) Carbon Core Photoluminescence: Carbon core photoluminescence refers to photon emission from carbon-based cores upon light absorption. As depicted in inset (IV) of Figure 1b, irradiation excites electrons from the ground state to excited states, which subsequently release photon energy upon returning to the ground state [10]. The luminescence of CDs results from the concerted contribution of multiple mechanisms rather than a single isolated process. These mechanisms mutually influence and constrain each other, synergistically regulating the luminescence properties of CDs, including tunable fluorescence emission, fluorescence quenching, and fluorescence quantum yield (QY), among other key aspects. In-depth research and an understanding of these synergistic mechanisms are of significant theoretical importance and practical value for the development of novel high-performance luminescent CD materials and their application technologies.

### 1.2. Biomass Derived Carbon Dots

As research on CDs progressed, carbon sources were found to critically influence CD properties and synthesis methodologies. Initial precursors mostly included fossil-derived macromolecular carbon (e.g., carbon black, graphite) [13], linear organic chemicals (e.g., polyvinyl alcohol) [14], and tiny aromatic molecules (such as aniline and phenolic structures) [15,16]. However, these precursors exhibit inherent limitations, including complex/expensive processing, nonrenewable availability, and poor scalability for industrial production. In contrast, B-CDs precursors originate from sustainable biological sources (e.g., cellulose, starch, and chitosan) and other biomass wastes/extracts (Figure 1c). These materials are renewable and biodegradable, mitigating environmental pollution from biological waste. CDs synthesized from biomass demonstrate exceptional photostability, low toxicity, and tunable optical properties. Consequently, propelled by ongoing research, B-CDs are now extensively deployed across diverse fields spanning biomedical applications [17], optoelectronic devices [18], fluorescence imaging [19], sensing technologies, photocatalysis, and drug delivery systems [20,21]. To deepen the understanding of biomass as carbon sources for CDs, this study systematically summarizes current advancements in the synthesis procedures, carbon precursors, formation processes, properties, and applications of B-CDs. First, primary preparation methods for B-CDs are introduced. Subsequently, the formation mechanisms and structural characterization of CDs derived from cellulose, starch, and chitosan are elaborated. The key photoluminescence (PL) properties, including tunable fluorescence emission, fluorescence quenching, and quantum yield, are then critically discussed. Current problems and prospective research directions for B-CDs are outlined. This study aims to provide theoretical insights for the advancement and use of B-CDs, therefore contributing to the Sustainable Development Goals.

## 2. Synthetic Methods

In recent years, the emergence of green chemistry and sustainable development has elevated the use of biomass resources, including cellulose, starch, and chitosan, as precursors of CDs [22]. CD synthesis methodologies are primarily categorized into top-down and bottom-up approaches. As summarized in Table 1, top-down methods involve the physical or chemical fragmentation of bulk carbon materials (e.g., graphite, carbon black) into nanosized CDs [23]. Common techniques include laser ablation, arc discharge, and oxidative acid treatment. These methods dominate graphite-derived CD production due to advantages such as controllable reaction conditions, straightforward protocols, and low material costs. However, the heterogeneous composition and structural irregularity of biomass impede uniform decomposition and etching during top-down processing, resulting in CDs with inconsistent size and morphology. Consequently, bottom-up synthesis prevails for B-CDs. As summarized in Table 2, bottom-up methods synthesize CDs incrementally from molecular precursors (e.g., tiny organic molecules, inorganic carbon sources) by carbonization, polymerization, or condensation processes [24]. In the bottom-up method, hydrothermal method can control the particle size and oxygen-rich surface groups well, but it usually takes several hours of reaction time and precise pH adjustment to avoid uncontrolled polymerization. Compared with this, microwave method can provide ultra-fast heating, short reaction time and high yield, but it is not good at size control. Compared with the first two methods, pyrolysis has the biggest advantage in that the synthesis process is simple, there is no need for solvents and high-pressure vessels in the synthesis process, and it also performs well in yield. However, pyrolysis consumes a lot of energy and produces less functional groups on the surface of B-CDs, and so post-treatment is needed to introduce oxygen-containing and nitrogen-containing parts needed for photoluminescence. Different synthesis methods have their own advantages and disadvantages in the synthesis of B-CDs, and are suitable for different precursors and application requirements. Therefore, researchers can choose a suitable bottom-up method to balance the quantum yield, fluorescence performance, and application requirements so as to sustainably produce high-performance and multifunctional B-CDs from biomass.

## 3. Raw Materials and Formation Mechanisms

Natural polymers serve as ideal carbon precursors for CD synthesis due to their carbon-rich composition and diverse chemical structures. They exhibit outstanding cost-effectiveness and inherent eco-friendliness, demonstrating intrinsic alignment with sustainable development paradigms. The comparison of carbon content and scalability of different biomass precursors is shown in Table 3. This chapter focuses on elucidating the formation processes and structural characterization of cellulose-, starch-, and chitosan-derived carbon dots.

### 3.1. Cellulose

#### 3.1.1. Introduction of Cellulose

**Figure 2:**
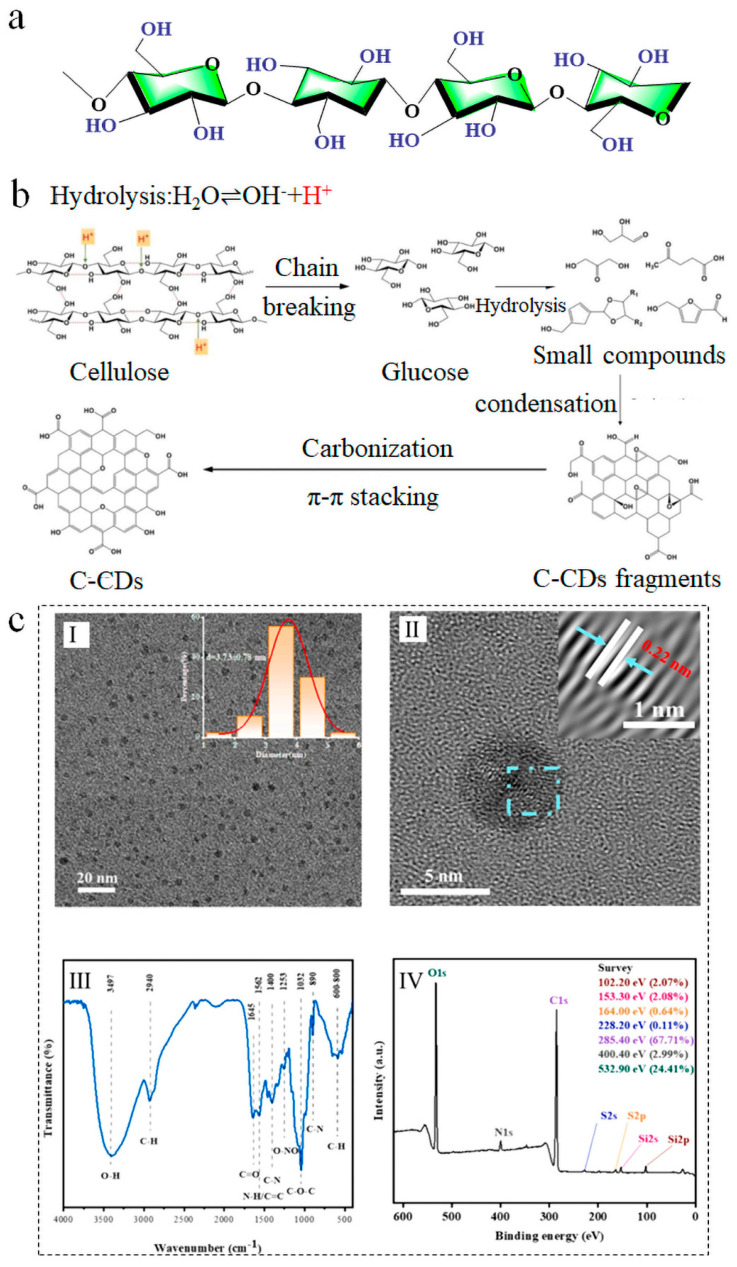
(**a**) Chemical structure of cellulose. (**b**) Cellulose chain breaking, hydrolysis, polymerization, and carbonization [34]. (**c**) The structural characterizations of SN-CDs are (**I**) size distribution, (**II**) TEM images, (**III**) FT-IR spectra, and (**IV**) XPS measurement spectra [25].

Cellulose, a polysaccharide characterized by its rigid molecular architecture and intermolecular hydrogen bonding, comprises D-glucose units connected by β-1,4-glycosidic bonds (Figure 2a) [32]. As one of nature’s most abundant renewable polymers, it is ubiquitously sourced from terrestrial flora. Cellulose exhibits exceptional reactivity in carbon dot synthesis owing to its high carbon content and profuse hydroxyl (-OH) groups [33]. The carbonization process demands significant energy input, and so cellulose-derived carbon dots (C-CDs) are usually produced by hydrothermal treatment or microwave irradiation. The profuse hydroxyl groups of cellulose provide reactive sites for CD formation, facilitating chemical bonding and structural reorganization during carbonization. Concurrently, cellulose’s innate linear rigidity promotes the formation of stable carbon frameworks, enabling uniform nucleation and the colloidal dispersion of CDs. Furthermore, its inherent biodegradability and hydrophilicity confer excellent biocompatibility and environmental safety to C-CDs, rendering them suitable for biomedical and environmental applications. These attributes collectively establish cellulose as a premier carbon precursor for high-performance CDs. Ongoing methodological innovations will continue to optimize the properties and expand the applicability of C-CDs.

#### 3.1.2. Cellulose-Based Carbon Dots

The formation mechanism of carbon dots (CDs) derived from cellulose primarily involves a multi-step process of structural deconstruction and carbon core assembly. As illustrated in Figure 2b, water first undergoes ionization under hydrothermal (high-temperature and high-pressure) or microwave conditions, generating H^+^ ions. Catalyzed by these H^+^ ions, the β-1,4-glycosidic bonds and hydrogen bonds within cellulose undergo cleavage under hydrothermal conditions. This degradation yields intermediate products such as glucose monomers and monosaccharides. Concurrently, as the reaction proceeds, small organic acid molecules are generated, further promoting molecular rearrangement and carbonization among the small intermediates. The initially formed monosaccharide intermediates subsequently undergo dehydration and deoxygenation reactions, generating carbon-rich intermediates characterized by conjugated structures. Under sustained high temperature or pressure, these intermediates undergo further polymerization and dehydration. Following rearrangement, they form graphitic microcrystalline cores (carbon nuclei) composed of sp^2^-hybridized carbon. These nuclei subsequently grow into carbon dots via π-π stacking and hydrogen bonding. Simultaneously, hydroxyl (-OH) and carboxyl (-COOH) groups originating from incompletely removed functional groups on the cellulose molecular chains form hydrophilic functional groups on the CD surface. These surface groups contribute significantly to their fluorescence properties. Crucially, the linear structures and hydrogen-bonding networks inherent to cellulose guide the orderly arrangement of the carbon nuclei. This templating effect reduces surface defects and ultimately facilitates the formation of CDs with uniform size and high crystallinity [34]. The fluorescence emission mechanism of these carbon dots mainly stems from the synergistic effect of quantum confinement and surface state emission.

Numerous instances illustrate that the morphology of carbon dots derived from biomass is often spherical or quasi-spherical, as shown by those from rice straw (Figure 2c) [25], wheat straw [35], cherry [36], tomato [37], and orange juice [38], among others. Yang et al. generated nitrogen/sulfur co-doped carbon quantum dots (SN-CQDs) from rice straw by a one-step hydrothermal process utilizing synergistic doping with lipoic acid and ethylenediamine [25]. Figure 2c presents the structural characterization of SN-CQDs. The TEM picture in the inset (I) of Figure 2c demonstrates that the carbon dots prepared from rice straw exhibit good dispersion, possess spherical structures, and contain particle sizes under 10 nm. HRTEM is used to ascertain the structure of the CDs through observing the spacing between the bright and dark fringes. This distance is then compared to standard crystal plane spacings to identify the specific crystallographic plane. The acquisition of clear lattice fringes in HRTEM indicates the good crystallinity of the B-CDs [39]. The inset (II) in Figure 2c shows an HRTEM image of SN-CQD, characterized by a lattice spacing of 0.22 nm. The FTIR of SN-CQD is shown in the inset (III) of Figure 2c. It can be seen that SN-CQD is rich in oxygen-containing functional groups, especially hydroxyl groups, carboxyl groups, ether bonds/alcohol hydroxyl groups, and alkyl chains, and possibly amino groups. The full XPS spectrum of SN-CQD is shown in the inset (IV) of Figure 2c. Peaks observed at binding energies of 285.4 eV, 532.9 eV, and 400.4 eV correspond to the C 1s, O 1s, and N 1s core levels, respectively. Their percentage compositions are 67.71%, 24.41%, and 2.99%. B-CDs typically contain carbon (C), oxygen (O), nitrogen (N), and sulfur (S), a composition that is reflected in their XPS spectra [25].

### 3.2. Starch

#### 3.2.1. Introduction of Starch

**Figure 3:**
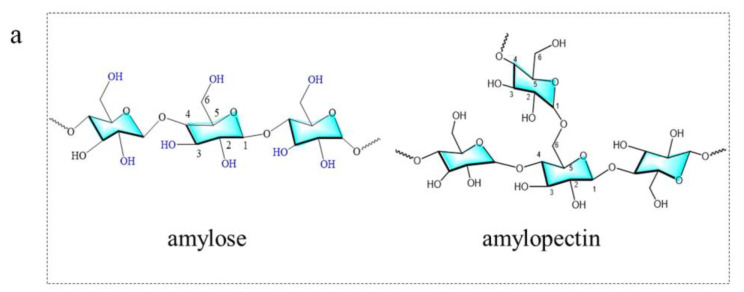

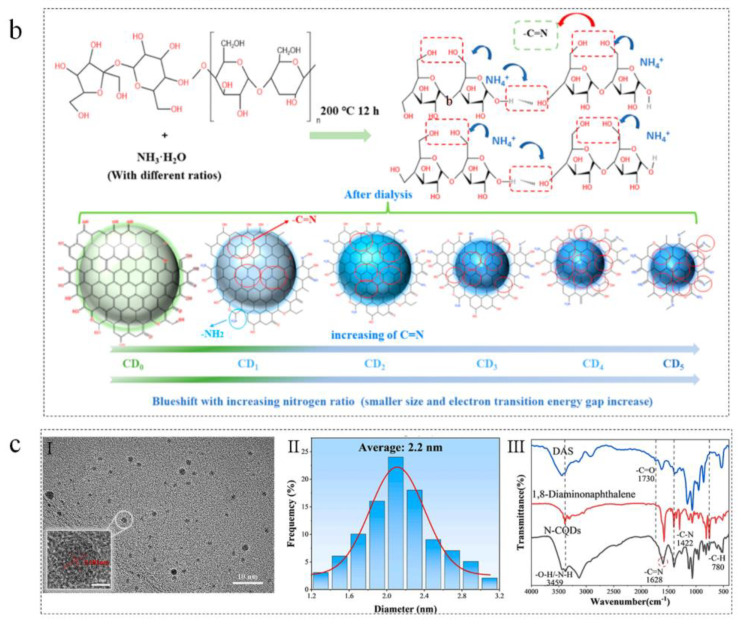
(**a**) Chemical structure of amylose and amylopectin. (**b**) Synthetic process of starch-based CDs with nitrogen doping [43]. (**c**) Structural characterization of N-CQDs: (**I**) TEM images and HRTEM images, (**II**) size distribution and (**III**) FT-IR spectra [26].

Starch serves as the principal energy storage medium in plants and is prevalent in crops like grains and potatoes. It is a polymer of glucose connected by α-1,4-glycosidic and α-1,6-glycosidic linkages, manifesting in two forms: amylose and amylopectin (Figure 3a) [40]. The carbon content of starch is similar to that of cellulose, but its molecular structure is more loosely packed, making its decomposition into smaller molecules at lower temperatures easier [41]. The synthesis of starch-based carbon dots (S-CDs) is typically carried out through hydrothermal or solvothermal methods, and they exhibit high water solubility and biocompatibility, rendering them appropriate for biomedical applications.

#### 3.2.2. Starch-Based Carbon Dots

The formation mechanism of starch-derived carbon dots is based on a dynamic process involving polysaccharide chain deconstruction and carbonaceous recombination. Initially, under hydrothermal/solvothermal conditions, long-chain starch molecules undergo hydrolysis, fragmenting into monosaccharides (e.g., glucose, maltose) or smaller organic fragments [42]. Subsequently, as illustrated in Figure 3b, the hydroxyl (-OH) and aldehyde (-C-H) groups of these monosaccharides react via dehydration reactions to form furfurals and other intermediates. These intermediates then undergo further carbonization and condensation under sustained high temperatures/pressures, generating carbon-rich structures that gradually aromatize to form carbon nuclei [43]. Ultimately, carbon dots are formed as the nuclei grow via fusion with carbonaceous fragments and become surface-passivated by oxygen-containing functional groups.

However, based on the understanding of the formation process of starch-based carbon dots, their structural characterization becomes a crucial step for gaining an in-depth understanding of their properties and applications. A series of advanced characterization techniques can comprehensively analyze their chemical composition, surface functional groups, crystalline structure, and morphological features. Liang et al. produced nitrogen-doped carbon quantum dots (N-CQDs) by a hydrothermal process using dialdehyde starch and 1,8-diaminonaphthalene. The TEM and HRTEM images in Figure 3c (I) show that the morphology of N-CQDs is spherical, exhibiting a lattice fringe spacing of 0.182 nm and possessing high crystallinity. The particle size distribution of N-CQDs, seen in Figure 3c (II), ranges from 1.2 to 3.2 nm, with an average size of 2.2 nm. The results indicated that the starch-based CDs exhibited excellent dispersibility. These results substantiate the presence of an intrinsic graphitic architecture inside the carbon cores of starch-derived carbon dots. FTIR spectra, as shown in inset (III) of Figure 3c, illustrate the structural composition of N-CQD. There is C=O stretching vibration of the aldehyde group in dialdehyde starch (DAS). However, N-CQD lacks this characteristic C=O peak. It is confirmed that 1,8-diaminonaphthalene is combined with the aldehyde group of DAS to synthesize N-CQD. Moreover, it can be seen from the figure that N-CQD surface is rich in-OH, -NH_2_ and aromatic C-N bonds, which enhance the hydrophilicity and stability of N-CQD [26].

### 3.3. Chitosan

#### 3.3.1. Introduction of Chitosan

**Figure 4:**
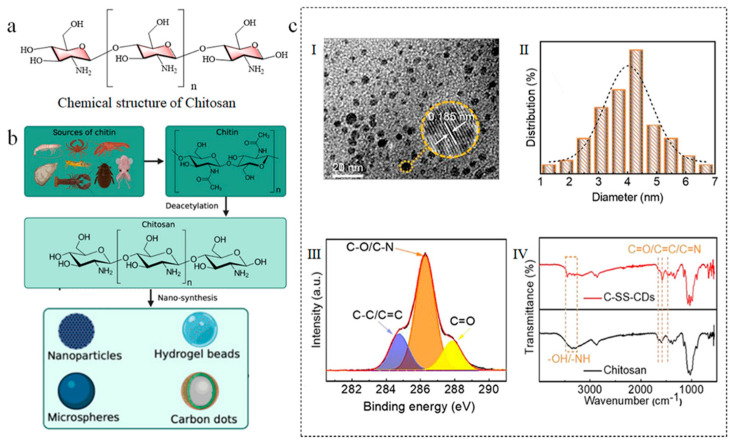
(**a**) Chemical structure of chitosan. (**b**) fabrication strategies for chitosan-derived carbon nanomaterials [47]. (**c**) Structural characterization of C-SS-CDs: (**I**) TEM images and HRTEM images, (**II**) size distribution, (**III**) XPS spectra, and (**IV**) FT-IR spectra [27].

Chitosan is a natural polysaccharide derived from the deacetylation of chitin and is widely present in the exoskeletons of crustaceans and fungal cell walls [44]. It consists of D-glucosamine and N-acetyl-D-glucosamine units, interconnected by β-1,4-glycosidic linkages and rich in amino (-NH_2_) and hydroxyl (-OH) groups (Figure 4a) [45]. Chitosan has a high carbon content, and its molecular structure contains nitrogen, which can enhance the fluorescence performance of carbon dots through intrinsic doping. Chitosan-based carbon dots (Ch-CDs) are typically synthesized through hydrothermal or microwave irradiation methods, and they exhibit excellent biocompatibility and fluorescence characteristics, suitable for applications in bioimaging and drug delivery.

#### 3.3.2. Chitosan-Based Carbon Dots

The preparation process of chitosan-derived carbon dots is illustrated in Figure 4b, where the raw material originates from chitosan obtained through the deacetylation of chitin that is biologically derived from crustaceans. The formation mechanism can be summarized as follows: Under hydrothermal/solvothermal conditions, chitosan molecules undergo deacetylation hydrolysis, releasing free amino groups and generating glucosamine fragments. Subsequently, amine-aldehyde condensation produces N-heterocyclic intermediates, which then undergo progressive carbonization and condensation to form N-doped carbon nuclei [46]. Ultimately, unreacted moieties self-assemble through hydrogen bonding and/or covalent interactions to form a self-passivation surface layer rich in functional groups, enabling in situ nitrogen doping and surface functionalization [47].

Chitosan, derived from the deacetylation of chitin, possesses free amino (-NH_2_) and hydroxyl groups, constituting a natural nitrogen-containing biopolymer. During carbonization, these amino groups directly form nitrogenous configurations such as pyridinic N and graphitic N, enabling intrinsic nitrogen doping that contributes to relatively high quantum yields. Nitrogen doping induces the formation of additional sp^2^ clusters, with the carbon core exhibiting intermediate structural ordering between that of cellulose-derived and starch-derived CDs. Ni et al. synthesized chitosan-derived carbon dots (C-SS-CDs) by a one-step hydrothermal process using chitosan and ethylenediamine. TEM images of C-SS-CDs reveal that the carbon dots possess a spherical morphology, as seen in inset (I) of Figure 4c, whereas HRTEM images indicate a lattice fringe spacing of 0.185 nm, demonstrating substantial crystallinity. The particle size distribution map in inset (II) of Figure 4c indicates that the average particle size of C-SS-CDs is 4.02 nm. XPS examination in inset (III) of Figure 4c reveals the existence of C-C/C=C, C-O/C-N, and C=O bonds at this carbon site. The FTIR spectra of C-SS-CDs shown in inset (IV) of Figure 4c demonstrate intensified tensile vibrations of -OH/-NH and C=C/C=O/C=N bonds, corroborating the XPS findings. FTIR research indicates that C-SS-CDs and chitosan precursors possess similar functional groups. This indicates that C-SS-CDs efficiently preserve the structural attributes of the chitosan molecular framework and exhibit structural similarities to chitosan [27].

## 4. PL Properties of Biomass CDs

B-CDs typically have a unique core–shell structure. The core is mainly a carbon framework with sp^2^/sp^3^ hybridization. The shell contains oxygen or nitrogen—containing functional groups bonded to the core. This complex and tunable chemical composition gives B-CDs excellent and diverse photoluminescence (PL) properties. The key PL features of B-CDs are tunable fluorescence emission, fluorescence quenching, quantum yield (a measure of their emission efficiency), and chiral carbon dots. The following is a detailed discussion of these key PL properties.

### 4.1. Tunable Fluorescence Emission

**Figure 5:**
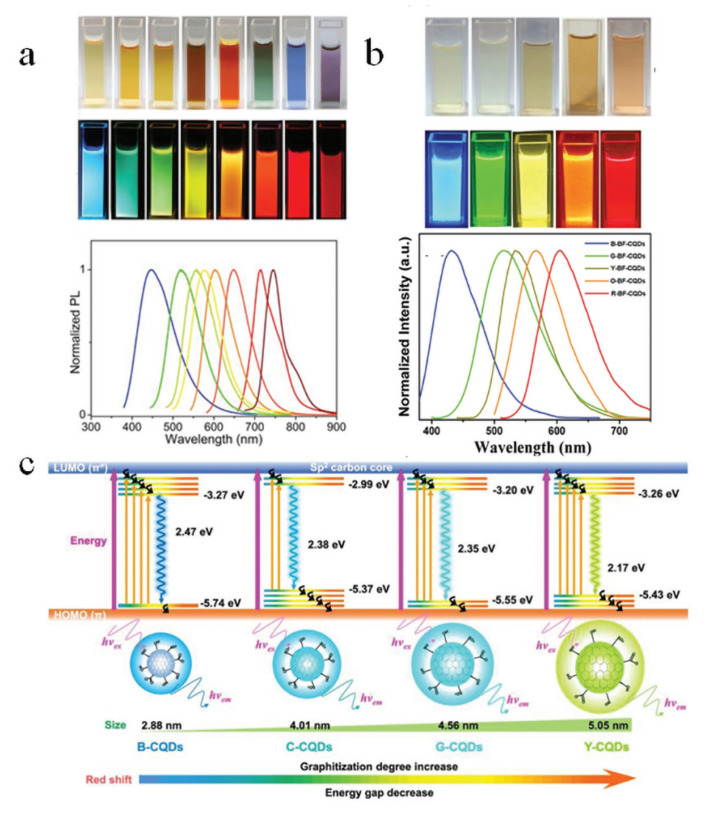

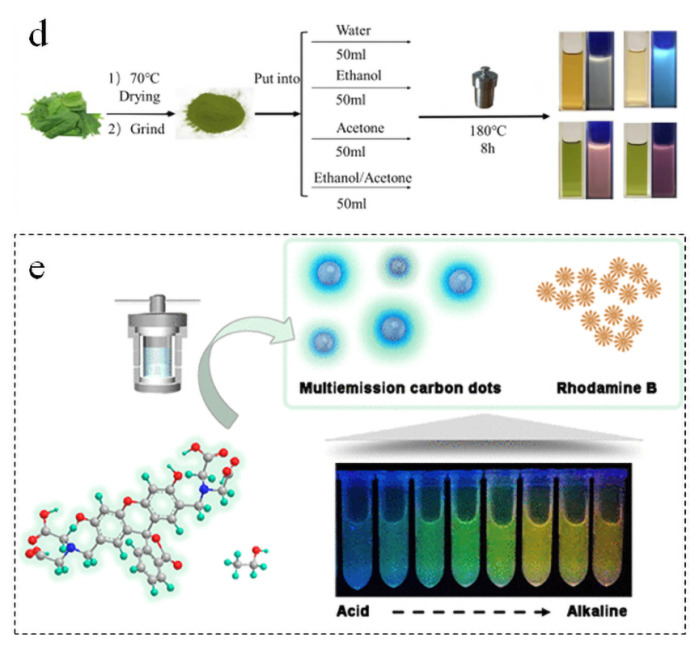
(**a**) Photographs of multicolor fluorescent CDs synthesized from L-glutamic acid and o-phenylenediamine under sunlight (upper layer) and ultraviolet light (lower layer). Normalized PL emission spectra of all samples under excitation at 365 nm [48]. (**b**) Photograph of polychromatic carbon dots under daylight (upper layer) and fluorescence image under ultraviolet light (excitation wavelength 365 nm) (lower layer) and normalized fluorescence spectrum under excitation at 365 nm [49]. (**c**) Size-dependent quantum confinement mediating redshift [50]. (**d**) Blue-emissive CD design for multicolor displays [51]. (**e**) Synthesis strategy for multiemission CDs (M-CDs) [52].

One of the most remarkable photoluminescent properties of B-CDs is their highly tunable fluorescence emission wavelength. This tunability primarily stems from their complex structural composition, which is highly susceptible to precursor materials and synthesis conditions. By changing the types of biomass precursors, dopants, preparation parameters and synthesis conditions, B-CDs with different wavelengths of fluorescence emission can be obtained. Ding et al. prepared a series of nitrogen-doped carbon dots with different fluorescence emission wavelengths by adjusting the reaction solvent of l- glutamic acid and o-phenylenediamine [48]. As shown in Figure 5a, these nitrogen-doped carbon dots show tunable PL emission from blue light (443 nm) to near-infrared (745 nm) positions under the excitation of 365 nm. The dopant used is also the decisive factor in regulating the fluorescence emission performance of B-CDs. Yuan et al. synthesized MCBF-CQDs from blue to red from CA and DAN (2,3- diaminonaphthalene or 1,5- diaminonaphthalene) by controlling heating time, solvent, and dopant type [49]. As shown in Figure 5b, the prepared MCBF-CQDs shows adjustable PL emission from blue light (430 nm) to red light (604 nm) positions under ultraviolet light (excitation wavelength is 365 nm). The regulation of fluorescence emission can also be achieved by changing the particle size of B-CDs based on the quantum size effect. As shown in Figure 5c, [50] the change in emission wavelength between CQD is caused by the gradual increase in particle size, graphitization degree, and SP carbon content. This enlarges the π conjugate range and reduces the band gap of HOMO-LUMO from 2.47 eV (blue) to 2.17 eV (yellow), thus inducing emission red shift. This trend emphasizes the key role of weakened quantum confinement effect and enhanced electron delocalization in luminescence modulation. Natural biomass often has complex components, and there will be self-doping. As shown in Figure 5d, Wang et al. used spinach as the sole carbon source and only synthesized four-color B-CDs by solvent tuning [51]. In this study, the carbonation process of polysaccharide and chlorophyll in spinach was changed by adjusting the solvent, and the regulation of B-CDs fluorescence emission was realized. As shown in Figure 5e, Zhu et al. prepared M-CDs from calcein [52]. The results show that the multicolor fluorescence emission of M-CDs is realized by the synergistic effect of solvents, surface defects, and precursor aromatic ring luminophores.

In summary, these studies clearly demonstrate that precise control over the core structure and surface states of B-CDs can be achieved through the careful selection of biomass precursors, the precise regulation of synthesis conditions, and the engineering of surface chemistry. This multifaceted fine-tuning strategy constitutes the core mechanism for endowing B-CDs with highly tunable fluorescence emission while synergistically optimizing their key optical properties. This advancement establishes a solid foundation for their practical applications in high-performance fluorescent sensing, advanced anti-counterfeiting encryption, and white light illumination and display.

### 4.2. Fluorescence Quenching Phenomenon

**Figure 6:**
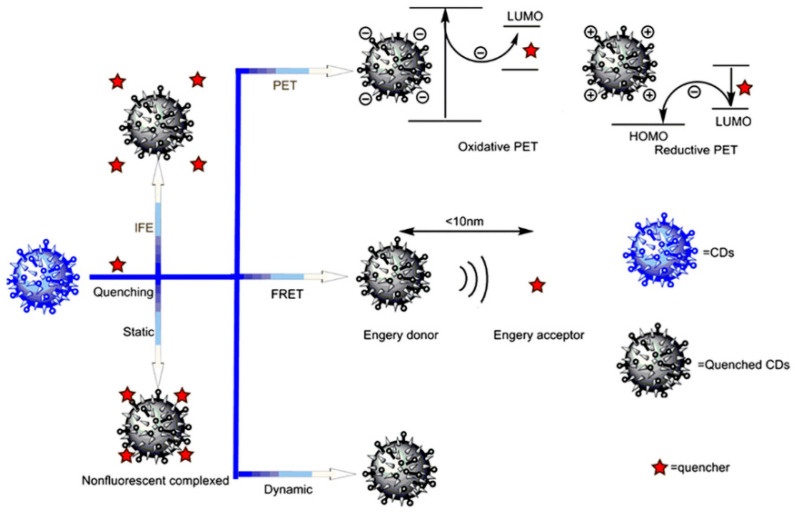
Quenching mechanisms of fluorescent CDs [53].

**Figure 7:**
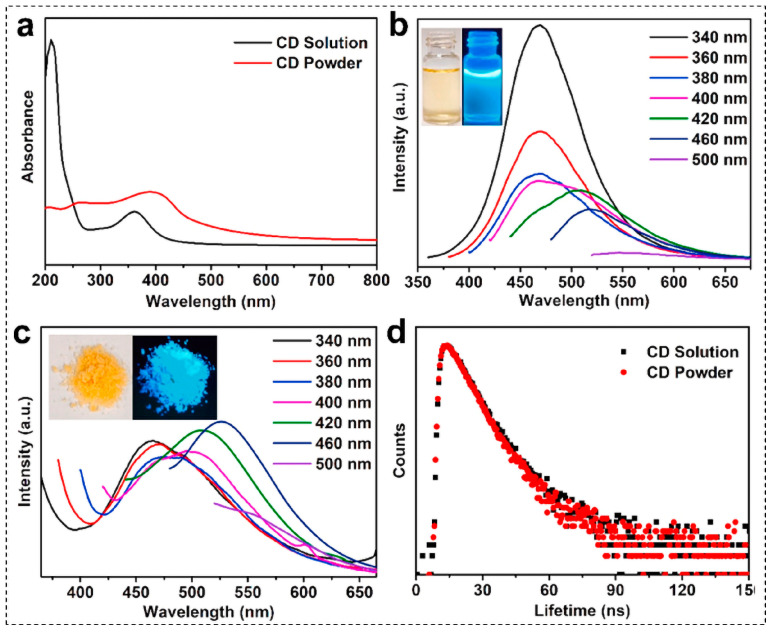
Characterization of optical properties of self-quenching-resistant N,P-doped carbon dots: (**a**) UV-vis absorption spectra of aqueous solutions and powders of CD; (**b**) Fluorescence emission spectrum of CD aqueous solution; (**c**) Fluorescence emission spectrum of CD powder; (**d**) Fluorescence attenuation curves of aqueous solutions and powders of CD (excitation wavelength 365 nm, emission wavelength 465 nm) [54].

The fluorescence quenching behavior of B-CDs represents a core manifestation of their high sensitivity to subtle changes in the external microenvironment, underpinning their utility as exceptional fluorescent probes. This quenching is primarily manifested as a significant reduction in or the complete disappearance of fluorescence intensity and can be triggered by diverse factors. These include specific metal ions, anions, small molecules, biomolecules (e.g., proteins, DNA), pH variations, alterations in redox environments, and temperature fluctuations. The underlying quenching mechanisms are complex and multifaceted, often involving processes such as photoinduced electron transfer (PET), Förster resonance energy transfer (FRET), static quenching, dynamic quenching, and the inner filter effect (IFE) (Figure 6) [53]. Current research on exploiting fluorescence quenching mechanisms for practical applications and development primarily focuses on two key strategies: (1) enhancing the quenching phenomenon towards specific target ions (e.g., for detection) through methods like heteroatom doping and (2) mitigating quenching effects to improve fluorescence stability via precursor selection optimization and surface passivation treatments. Heteroatom doping can change the electronic structure, emission stability, and quenching resistance of B-CDs. Sujie Han et al. designed a novel anti-self-quenching N, P-doped CD (Figure 7a–d) [54]. The research shows that the surface entanglement of N/P-doped polyethyleneimine (PEI) chains can form a steric barrier between carbon cores, preventing self-quenching caused by π-π stacking, improving the emission stability of B-CDs, and thus maintaining bright emission for a long time in the solid state. Madhusudan Kr. Mahto et al. reported a conceptual framework based on molecular fluorophores to explain the quenching mechanism of blue-emitting nitrogen-doped carbon dots (N-CDs) [55]. The research shows that nitrogen doping provided by urea in N-doped carbon dots (NCDs) not only generates molecular fluorophores and intermediate energy levels at the core and surface of carbon dots, reduces the band gap and improves the quantum yield, but also provides favorable conditions for FRET and PET by optimizing the energy level layout and enhancing the spectral overlap, thus significantly improving the fluorescence quenching sensitivity and selectivity of carbon dots to target molecules.

In summary, the unique fluorescence quenching behavior of B-CDs represents their fundamental property as highly sensitive fluorescent probes, enabling them to respond sensitively to diverse environmental stimuli. A profound understanding and precise modulation of their complex quenching mechanisms are not only critical for developing high-performance sensors targeting specific analytes but also essential for improving the fluorescence stability of the carbon dot materials themselves. Current research, spanning from material design to mechanistic elucidation, is continuously deepening understanding and optimizing performance. This progress is driving the advancement of sophisticated sensing and photo-functional materials based on the fluorescence quenching properties of B-CDs.

### 4.3. Quantum Yield

Quantum yield (QY), defined as the ratio of emitted to absorbed photons under monochromatic excitation, serves as a fundamental metric for evaluating the photoluminescence efficiency of B-CDs, directly reflecting their photon utilization capacity. To enhance QY, critical strategies focus on defect passivation and luminescent center optimization: (1) selecting heteroatom-rich (N, S) biomass precursors enables intrinsic self-doping during carbonization to create favorable emissive states; [56] (2) utilizing heteroatom doping (N, S, P) allows us to enhance the transition of B-CDs’ surface state excitons to the ground state; (3) synthetic condition modulation optimizes carbon core crystallinity and surface chemistry. Through the systematic implementation of these approaches, QY can be substantially enhanced, establishing a robust foundation for practical applications [57]. Representative QY values of B-CDs derived from diverse carbon sources and heteroatom doping configurations are summarized in Table 4. The quantum yield of B-CDs is controlled by the choice of precursors, the type and concentration of dopants and the preparation parameters. At present, the quantum yield of biomass carbon dots is between 11.3% and 84.9%. Therefore, in order to obtain biomass carbon dots with high quantum yield, it is necessary to further study the interaction mechanism between these key factors and optimize the synthesis strategy in the future.

### 4.4. Chiral Carbon Dots

**Figure 8:**
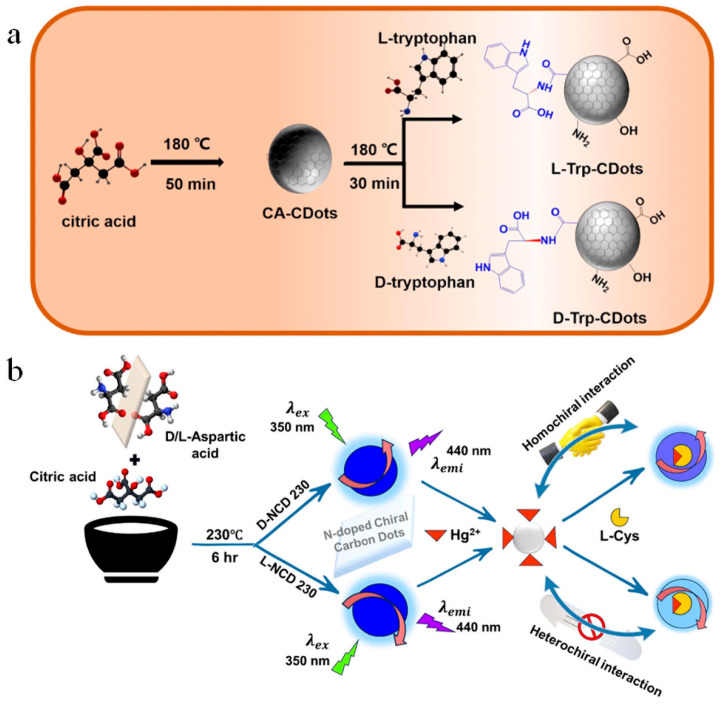
(**a**) Synthesis scheme of chiral carbon dot [64]. (**b**) Schematic diagram of preparation of N-doped chiral carbon dot and interaction between N-doped chiral carbon dot and Hg^2+^ [65].

In the field of optical properties of B-CDs, chiral carbon dots are expected to be endowed with more unique functions, especially in chiral recognition, biological imaging, and circularly polarized light emission, by introducing chirality to molecules or nanostructures. Chirality refers to the property that a molecule or nanostructure does not overlap its mirror image in spatial configuration [62]. By introducing chirality in molecules or morphology, chiral carbon dots not only retain the chirality of precursors, but also exhibit highly symmetric chiral optical properties, and have chemical stability, antibacterial, anti-tumor and other characteristics, giving carbon dots wider application potential [63]. In this respect, many studies focused on the synthesis using chiral precursors such as amino acids or glycans. Wang et al. obtained chiral carbon dots by two-step thermal polymerization using L-/D-tryptophan (Trp) as chiral source and citric acid as precursor (Figure 8a) [64]. Bhattacharya et al. synthesized nitrogen-doped chiral carbon dots (NCDs), specifically D-NCD230 and L-NCD230, by the thermal pyrolysis of citric acid and D/L-aspartic acid (Figure 8b). These chiral carbon dots allow the detection of Hg^2^ ion and L-cysteine by fluorescence off/on system. Their study showed that D-NCD230 exhibits stronger binding to Hg^2^ ion, resulting in lower detection limits. The fluorescence intensity of D-NCD230 is restored after the addition of L-cysteine. This work emphasizes the role of chirality in enhancing the optical asymmetry of selective sensing applications [65]. By introducing chiral carbon dots, it not only enhances the optical properties of B-CDs but also further broadens their applications in fields such as nanomedicine and environmental monitoring. Application: Although chiral carbon dots show significant advantages in the preparation of biomass carbon dots, the transition from laboratory-scale use to industrial production still faces challenges. Therefore, future research will focus on optimizing the synthesis process of chiral carbon dots and improving their application performance in biological and industrial fields.

### 4.5. Room-Temperature Phosphorescence

**Figure 9:**
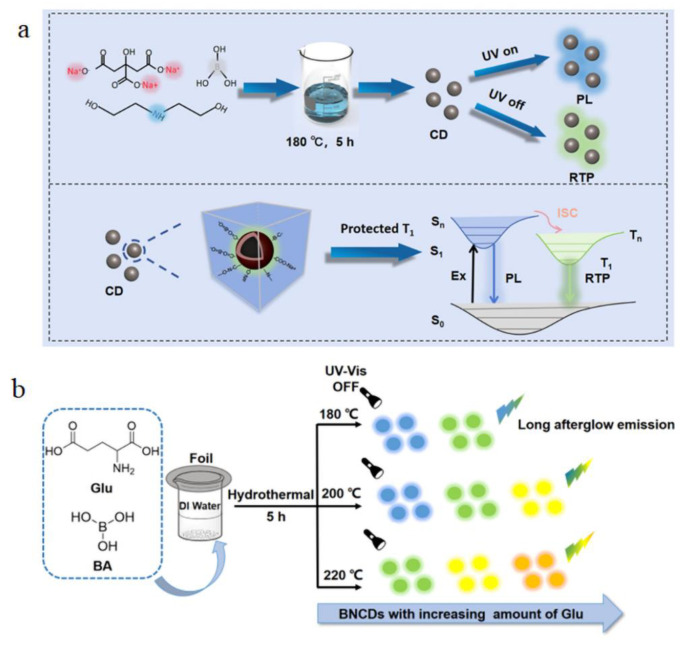

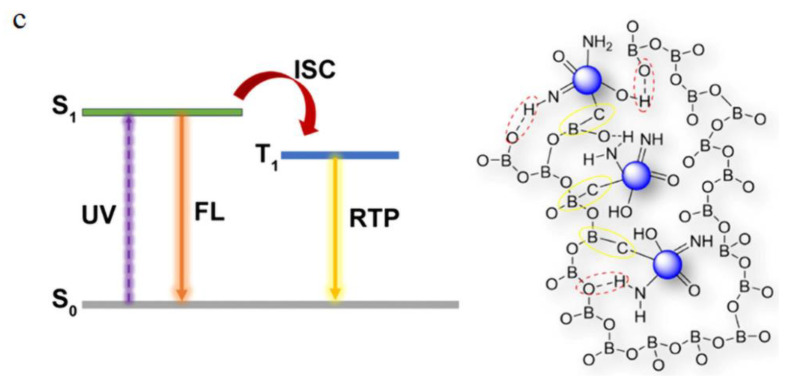
(**a**) Preparation method of BNCD@Na and RTP mechanism of BNCD@Na [68]. (**b**) Schematic diagram of preparation and emission characteristics of BNCD. (**c**) Schematic diagram and structure diagram of possible RTP emission process of BNCD [69].

Room-temperature phosphorescence (RTP) is an important and novel luminescence phenomenon in the framework of the optical properties of B-CDs. RTP emission is characterized by a long afterglow lasting milliseconds to seconds at room temperature through a radiative transition from triplet to ground states [66]. Compared with fluorescence, RTP emission has a longer lifetime and a larger Stokes shift, which makes it easier to separate from background fluorescence and enables high signal-to-noise ratio detection [67]. Heteroatom doping is usually a common and effective strategy in the performance tuning of biomass carbon dots, including room-temperature phosphorescence. Hu et al. synthesized blue fluorescence and blue-green afterglow B, N, Na-doped CDs using the hydrothermal method (Figure 9a). Sodium (Na)-doped carbon dots showed long phosphorescence lifetime in B_2_O_3_ matrix. The room-temperature phosphorescence intensity and phosphorescence lifetime of carbon dots increase after Na doping, because Na doping enhances intramolecular hydrogen bonds and covalent bonds, suppresses nonradiative transitions, and thus prolongs phosphorescence lifetime. In addition, the rigid structure of B_2_O_3_ host further suppresses nonradiative decay by restricting the molecular motion of carbon dots, thus promoting the stability and intensity of room-temperature phosphorescence [68]. In Sun et al.’s study, nitrogen (N)- and boron (B)-doped biomass carbon dots (BNCDs) exhibit tunable multicolor room-temperature phosphorescence (Figure 9b). By adjusting the reaction temperature and the amount of precursors, BNCDs can achieve a dynamic color change from yellow to green, and their room-temperature phosphorescence lifetime can reach 13 s. The results show that the reason for the efficient RTP emission of BNCDs is closely related to the rigid host B_2_O_3_ and C-B covalent bonds, which suppress nonradiative transitions such as vibration, rotation and collision, which may lead to triplet exciton quenching, thus producing efficient RTP. Meanwhile, C=O bonds play an important role in RTP color modulation as key emission centers. Through the RTP mechanism and XPS analysis of BNCDs, the RTP mechanism and structure of BNCDs are predicted (Figure 9c) [69]. Although many achievements have been made in room-temperature phosphorescence, there are still many challenges in the research of room-temperature phosphorescence of B-CDs. For example, the stability of room-temperature phosphorescence of B-CDs under water and temperature changes still needs to be further optimized. The studies of Hu et al. and Sun et al. showed that water and thermal stimuli can affect the phosphorescence characteristics of B-CDs. Moreover, the large-scale production of biomass carbon dots is also a difficult problem to solve. Future research should focus on simplifying the synthesis process, reducing costs, and exploring applications of these materials in a wide range of fields, such as biomedicine, environmental monitoring, etc. Overall, despite some technical challenges, the room-temperature phosphorescence and multicolor tunability of biomass carbon dots make them promising for information encryption, anti-counterfeiting, and other high-tech fields.

## 5. Functional Applications

**Figure 10:**
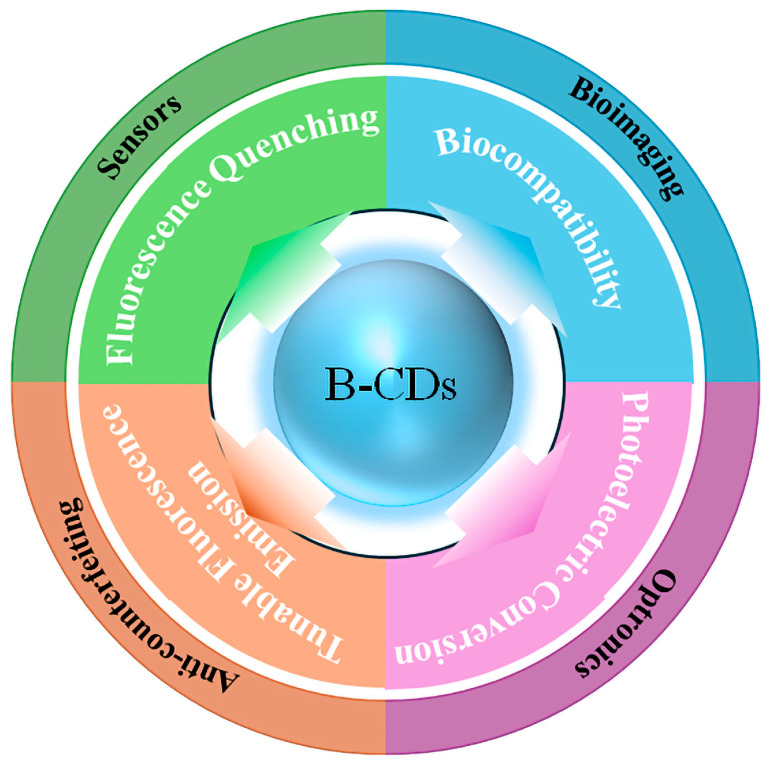
Schematic diagram of various applications of B-CDs.

CD synthesized from biomass has unique structural characteristics such as adjustable carbon core size and graphitization degree, heteroatom-rich surface functional groups, and diverse surface/molecular fluorophores, which give it key functions in multi-field applications. As shown in Figure 10, in the sensor field, B-CDs have unique fluorescence quenching or enhancement effects, which can achieve high sensitivity and high selectivity ion detection. In the field of anti-counterfeiting, B-CDs can display tunable fluorescence emission by adjusting the core–shell structure and surface state, realizing a dynamic “optical code”. In the field of biological imaging, hydrophilic functional groups such as carboxyl and amino groups on the surface not only improve the dispersion and biocompatibility of the aqueous phase but also label antibodies and peptide chains by covalent or electrostatic methods to achieve targeted imaging. Finally, in the field of optoelectronics, B-CDs can be used as high-efficiency electroluminescent layers or photosensitive active layers. Their broad tunable emission and excellent charge injection/transport characteristics significantly improve the performance and stability of LEDs and solar concentrators (LSCs). It is the unique structural characteristics of B-CDs that make them exhibit excellent versatility and customization advantages in the fields of sensors, anti-counterfeiting, bioimaging, and optoelectronics.

### 5.1. Sensors

**Figure 11:**
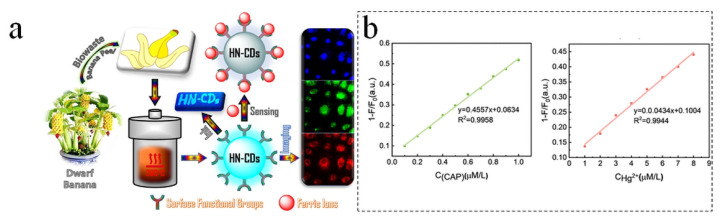

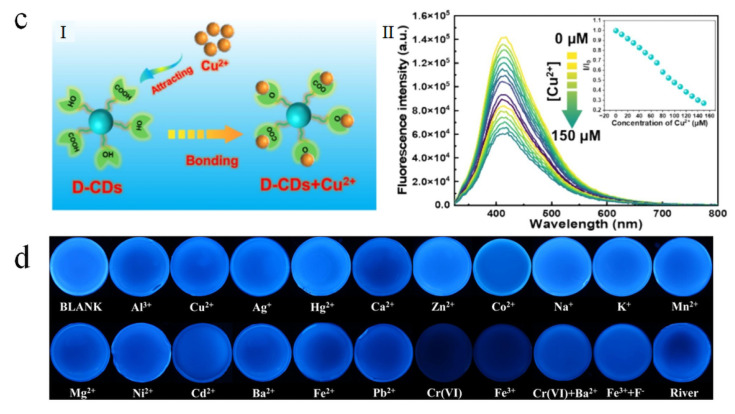
(**a**) Synthesis process and application schematic diagram of dwarf banana peel fluorescence HN-CDs [70]. (**b**) Standard curve of B-CDs for CAP and Hg2+ detection [71]. (**c**) Mechanism of D-CDs as sensor and detection performance for Cu^2+^: (**I**) binding mechanism of D-CDs with Cu^2+^; (**II**) fluorescence spectra of D-CDs with different Cu^2+^ (0–150 μM) content. Inset: Scatter plot of fluorescence intensity of D-CDs with increasing Cu^2+^ concentration [72]. (**d**) Response of FW-CD to various metal ions. Concentration of each metal ion was 100 μM [73].

As carbon dots have unique photoluminescence, their quenching phenomenon can be applied to respond to the variation in metal ion concentration. This feature of B-CDs enables the highly sensitive and selective detection of trace chemicals in environmental monitoring and sensor technology, crucial for early pollution warnings and control. Additionally, their renewability and reusability reduce environmental pollution and costs. B-CDs demonstrate exceptional capabilities for high-sensitivity metal ion detection. Atchudan et al. developed nitrogen-doped CDs from dwarf banana peel waste, leveraging specific chelation between surface carboxyl/phenolic hydroxyl groups and Fe^3+^ ions (Figure 11a). This sensor achieved >95% recovery rates in complex water matrices, validating B-CDs’ utility in environmental monitoring [70]. Feng et al. synthesized CDs via xylose carbonization, whose surface defect engineering enhanced electron-transfer efficiency, enabling selective Cu^2+^ detection with a 0.64 μM limit of detection (LOD) and 94.3% accuracy in Lake Tai water samples (Inset I to II in Figure 11c) [71]. For multi-target sensing, Liu et al. synthesized sulfur/nitrogen-co-doped blue-emissive CDs (S, N-B-CDs) from tartaric acid and 1-amino-2-naphthol-4-sulfonic acid. They established a dual-mode Hg^2+^/chloramphenicol (CAP) detection system employing Hg^2+^-induced static quenching and CAP-triggered π-π stacking fluorescence recovery, achieving 80.0–110.0% recovery in serum with low relative standard deviation (Figure 11b) [72]. Xia et al. utilized wintersweet flower waste to prepare green-emissive CDs for the parallel semi-quantitative visual detection of Cr(VI) and Fe^3+^. Through dual-masking strategies, mutual interference was eliminated, enabling independent quantification across distinct concentration ranges (Figure 11d) [73]. These studies show that the unique fluorescence quenching or enhancement effect of B-CDs can realize high-sensitivity and high-selectivity ion detection, so they are widely used in the field of sensors. Although B-CDs show excellent sensitivity and selectivity in metal ion sensing, they still face several challenges in practical applications: firstly, the precursors derived from natural biomass are often complex and have large differences between batches, which makes it difficult to reproduce the fluorescence performance and surface functional group distribution of carbon dots; secondly, in the face of complex real environments, long-term illumination or organic substances and coexisting ions in the environment may affect the sensing stability; finally, large-scale preparation and purification processes are not yet fully industrialized, and preparation costs and yields remain unresolved. Overall, these studies suggest that B-CDs can be used as sensors for detecting multiple metal ions, while double masking enables the parallel detection of two ions to adapt to complex environments in reality. However, there are still many problems to be solved in the practical application of B-CDs in the field of sensors. Future research should focus on unified precursor treatment, reductions in environmental interference, and large-scale industrial production to promote the development of B-CD sensors towards industrialization and real-time on-site monitoring.

### 5.2. Anti-Counterfeiting

**Figure 12:**
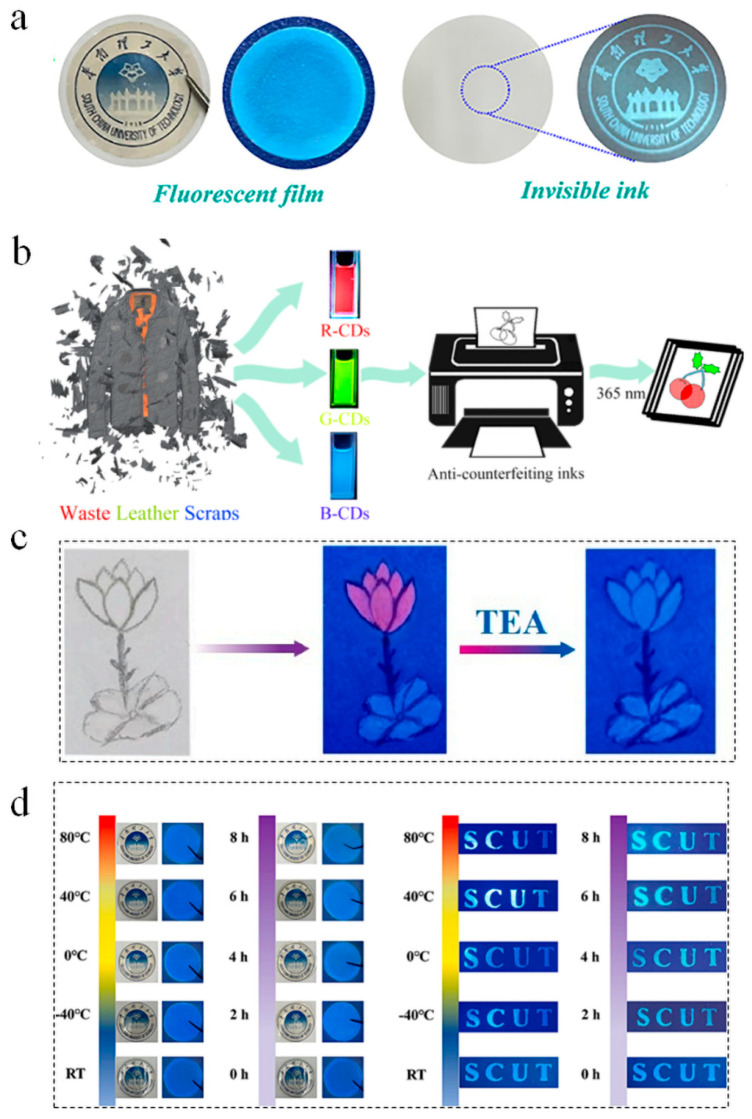
(**a**) Schematic diagram of N, S/P co-doped hemicellulose-based carbon dots for anti-counterfeiting applications [74]. (**b**) Schematic diagram of synthesis and anti-counterfeiting applications of multicolor carbon dots from waste leather scraps [75]. (**c**) Pattern anti-counterfeiting process based on spirulina derived carbon dots [76]. (**d**) Different temperatures of films and printed characters under UV (365 nm) light irradiation (RT, −40 °C, 0 °C, 40 °C, 80 °C) and different aging times (0, 2, 4, 6, 8 h). RT stands for “room temperature” [77].

In the field of anti-counterfeiting, the excellent photochemical stability of B-CDs ensures the durability of markings. Their low cost and sustainable nature facilitate large-scale production, while low toxicity and biodegradability guarantee environmental and human safety. Furthermore, their ease of integration and customization provides adaptable anti-counterfeiting solutions tailored to diverse products. Gao et al. synthesized carbon dots (CDs) from hemicellulose waste generated during papermaking via hydrothermal methods (Figure 12a). Through nitrogen, sulfur, and phosphorus doping, tunable fluorescence emissions (bright blue, dark cyan, and light cyan) were achieved. These CDs were successfully incorporated into cotton fibers to create anti-counterfeiting inks and films, demonstrating their potential for security marking and document protection [74]. Wenbo Zhang et al. utilized waste leather to fabricate red/green/blue tricolor CDs by solvent polarity-mediated emission tuning (Figure 12b). These CDs were engineered into anti-counterfeiting inks and flexible films exhibiting chemoresponsive luminescence color shifts, thereby enhancing security protocols [75]. Separately, Lina Zhang et al. developed dual-emissive CDs from spirulina precursors via a facile hydrothermal route, which displayed pH-responsive fluorescence at 456 nm and 677 nm. Dynamic emission switching enabled reversible data encoding/decryption, rendering them highly suitable for anti-counterfeiting and information security systems (Figure 12c) [76]. Guo et al. further expanded this domain by converting industrial lignin waste into nitrogen-doped lignin carbon dots (N-LCDs) through solvothermal synthesis. The N-LCDs exhibited a high-photoluminescence quantum yield of 42.69%, stable blue emission, and extreme environmental tolerance. They were developed into 1200 dpi high-precision anti-counterfeiting inks for fluorescent labels, providing a cost-effective (vs. rare-earth materials) and biodegradable solution for sustainable security applications (Figure 12d) [77]. In these studies, B-CDs can realize its unique tunable fluorescence emission by adjusting the core–shell structure and surface state, which makes it applicable to anti-counterfeiting field. With the development of this technology, B-CDs have shown great practical value in product traceability, intellectual property protection, and anti-counterfeiting marking, highlighting their broad market potential. B-CDs have shown great potential in the field of anti-counterfeiting, providing a sustainable, low-cost, and highly adjustable development idea for information encryption and storage. Their tunable fluorescence properties, photochemical stability and environmental friendliness make them a promising material for creating safe, durable and difficult-to-replicate labels on a wide range of products. However, there are still many shortcomings and challenges in the application of B-CDs in the anti-counterfeiting field, such as the fracture resistance and complexity of anti-counterfeiting patterns still need to be improved. Future research should focus on clarifying the impact of various biomass precursors; optimizing synthesis and post-processing processes; improving batch-to-batch consistency; developing anti-counterfeiting strategies to enhance anti-counterfeiting capabilities; and investing more in large-scale production to ensure that B-CDs anti-counterfeiting technology achieves real breakthroughs in reliability, scalability, and sustainability.

### 5.3. Bioimaging

**Figure 13:**
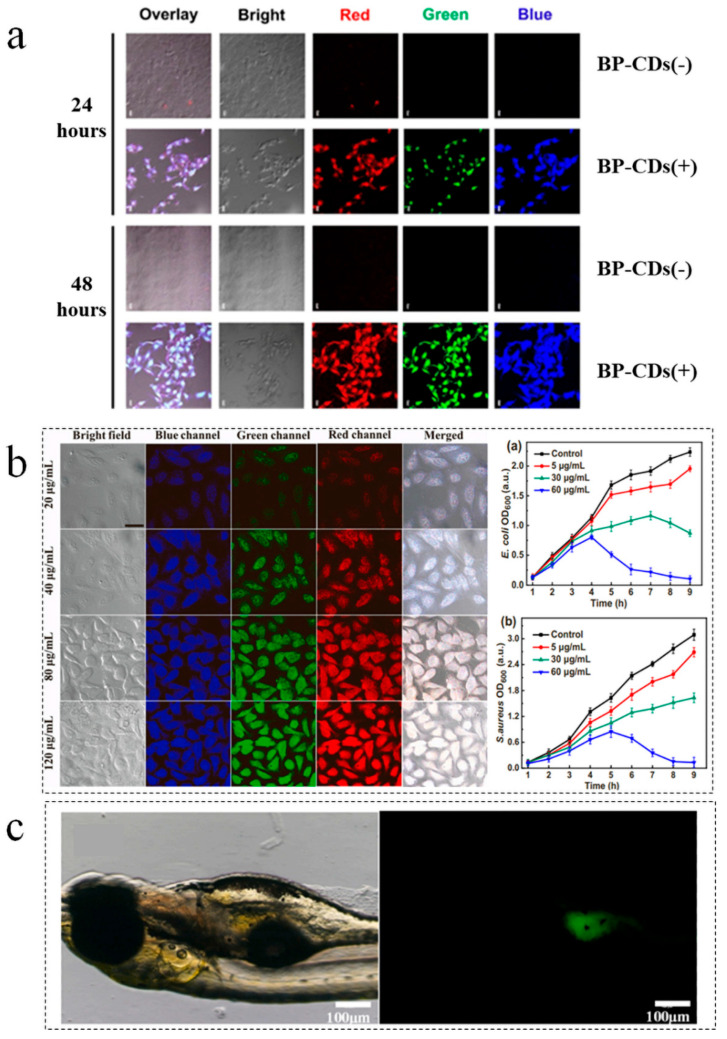
(**a**) Fluorescence imaging of BP-CD-labeled thyroid cancer [78]. (**b**) CLSM fluorescence images of HeLa cells incubated with different concentrations of N, P-CDs at a scale of 20 μm [80]. (**c**) Whole-body green fluorescence imaging of zebrafish 24 h post-injection with 100× diluted FS-CD solution [81].

B-CDs exhibit significant advantages in bioimaging owing to their low cytotoxicity. This property enables the long-term retention of fluorescence characteristics in vivo without eliciting significant immune responses. Their fluorescence color can be tuned by adjusting the excitation wavelength, facilitating multicolor, and deep-tissue imaging capabilities. Atchudan et al. synthesized nitrogen-doped carbon dots (BP-CDs) from banana peel waste via hydrothermal carbonization, exhibiting a uniform size of 5 nm, 19% quantum yield, and 120-day stability. As illustrated in Figure 13a, these BP-CDs enabled the direct labeling of human thyroid and breast cancer cells without modification, facilitating high-contrast fluorescence imaging for cost-effective cancer cell tracking [78]. Thota et al. utilized groundnut shells to fabricate CDs through hydrothermal carbonization, achieving 17.1% quantum yield and demonstrating excitation-dependent multicolor emission, including upconversion luminescence. Successfully applied in live yeast cell imaging, their low cytotoxicity supports real-time noninvasive bioimaging [79]. Hua et al. employed mango peel precursors to develop N, P-co-doped CDs (N, P-CDs) via microwave synthesis, featuring tunable multicolor fluorescence and negatively charged surfaces. As illustrated in Figure 13b, these simultaneously enabled multicolor cellular labeling and synergistic antibacterial activity against E. coli and S. aureus, establishing a dual imaging/therapeutic strategy for infected lesions [80]. Liu et al. engineered fish scale-derived CDs (FS-CDs) via DMF-mediated solvothermal synthesis, attaining an exceptional 31.71% quantum yield and excitation-tunable emission at 510 nm (Figure 13c). FS-CDs demonstrated high signal-to-noise ratios and minimal tissue autofluorescence interference during zebrafish in vivo imaging, confirming their suitability for deep-tissue applications [81]. In these studies, hydrophilic functional groups such as carboxyl and amino groups on the surface of B-CDs improve the dispersibility and biocompatibility of the aqueous phase, and B-CDs have low cytotoxicity. These characteristics enable it to retain its fluorescence characteristics in vivo for a long time without causing significant immune response. Moreover, B-CDs have unique optical properties of tunable fluorescence emission, and so B-CDs can realize multicolor and deep tissue imaging capabilities in the field of biological imaging. However, despite these remarkable advantages, B-CD still faces many challenges in practical biological imaging applications. For example, the fluorescence intensity attenuation under the long-term excitation of B-CDs affects the continuity and stability of imaging. Moreover, the complex physiological environment in the body may also interfere with the signal of B-CDs. In view of these problems, future research should focus on the standardization of synthesis process, the enhancement of photostability, and the elimination of interference from biological factors so as to truly promote the wide application of B-CDs in the field of biological imaging.

### 5.4. Optronics

**Figure 14:**
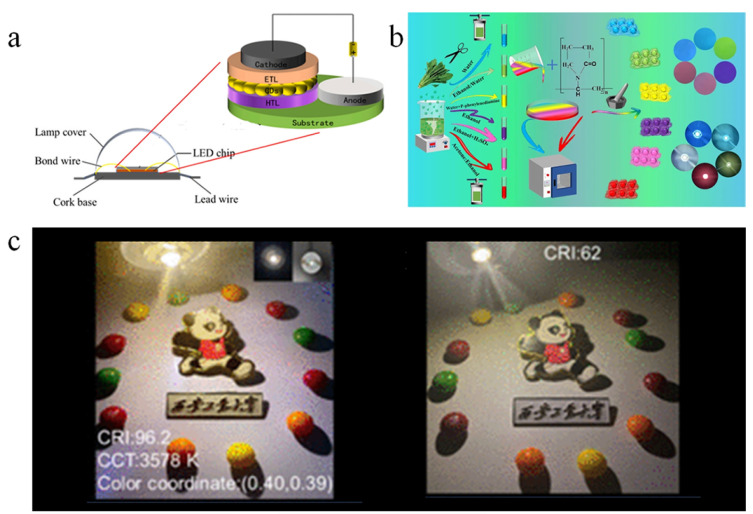

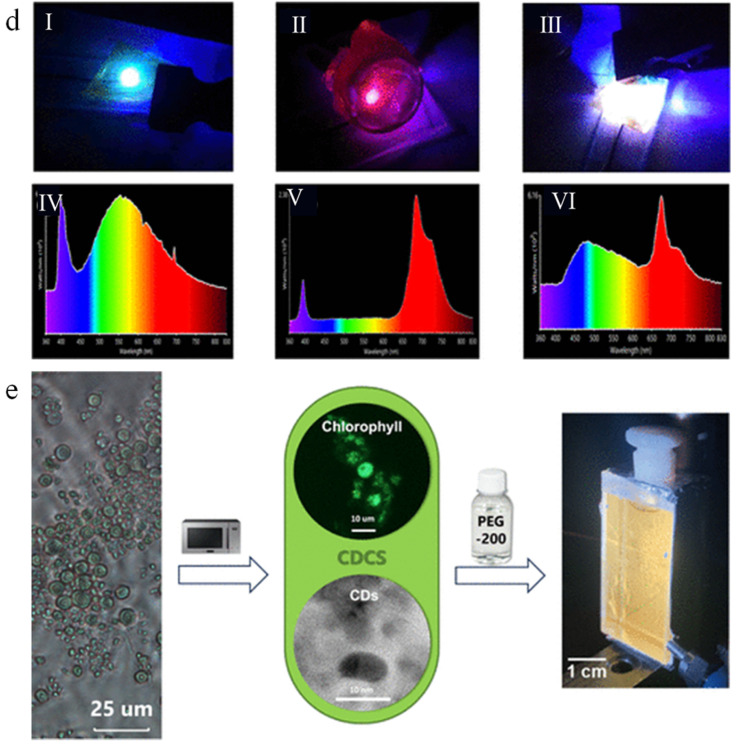
(**a**) The structure diagram of electroluminescent LEDs [82]. (**b**) The schematic of the fabrication process for the W-LED, operational image, electroluminescence (EL) spectrum, and corresponding CIE chromaticity diagram [83]. (**c**) The synthesis diagram of multicolor B-CDs for LED fabrication [84]. (**d**) Composite polymer films of CDs after excitation of blue LED chips: (**I**) G-CDs/PVA; (**II**) R-Cds/Pu; (**III**) white LEDs. (**IV**–**VI**) Electroluminescence (EL) spectra of G-CDs/PVA films, R-CDs/PU films and WLEDs [85]. (**e**) A schematic of the novel carbon dot/chlorophyll (CDCS) system derived from chlorella for application in LSCs [86].

Regarding the field of optoelectronics, B-CDs can achieve tunable photoluminescence from blue to red over the entire visible spectrum by adjusting the precursor type or reaction conditions. These features enable their direct integration into electroluminescent LED devices (Figure 14a) [82]. For instance, Ashok Kumar et al. synthesized red-emissive CDs from plant leaves and applied them in white LEDs (Figure 14b), achieving a high color rendering index (CRI) of 97, indicating strong potential for high-quality lighting [83]. Haozhe Wang et al. developed full-color CDs with high-photoluminescence quantum yields (PLQYs) from modified Zanthoxylum seeds, which were used to fabricate warm white LEDs (Inset I to II in Figure 14c) with a CRI of up to 96.2, while also demonstrating extended functionality in optical encryption applications [84]. Similarly, Shipeng Wang et al. utilized spinach-derived CDs and a solvent-controlled synthesis strategy to achieve multicolor emissions and fabricate LED devices with broad CIE coordinate coverage (Inset I to VI in Figure 14d), highlighting their applicability in both display and lighting technologies [85]. Beyond LED applications, B-CDs can also be applied to luminescent solar concentrators (LSCs). Filipe M. Santos et al. developed a novel carbon dot/chlorophyll (CDCS) system derived from Chlorella pyrenoidosa and applied it to LSCs (Figure 14e). This system exhibited significant photoelectric conversion efficiency and photostability under simulated sunlight irradiation [86]. In these studies, B-CDs, as an efficient electroluminescent layer or photosensitive active layer, have significantly improved the performance and stability of LED and solar concentrator (LSC) via their wide tunable emission and excellent charge injection/transmission characteristics. In general, B-CDs can achieve tunable luminescence in the full visible spectrum by selecting precursors and adjusting reaction conditions, and can be directly used in electroluminescent LED devices to achieve various functions such as illumination, display, and encryption. In addition, their excellent photoelectric conversion efficiency can also be used to improve the efficiency of LSC. However, in the practical application of photoelectric field, B-CD still faces many shortcomings and challenges. For example, almost all CD-derived LEDs constructed so far will have a certain degree of light (that is, blue or ultraviolet) leakage when a sufficiently high voltage is applied, which may cause damage to the human retina. Therefore, future research directions should focus on synthesis process optimization and standardization, photoelectric performance improvement, and large-scale synthesis process optimization, so as to promote the wider application of B-CDs in the photoelectric field and promote the development of sustainable photoelectric technology.

## 6. Conclusions and Outlook

Compared to traditional carbon dots, B-CDs are prepared from green carbon sources that are inexpensive and easily available from biomass waste, reducing dependence on fossil fuels. B-CDs are rich in oxygen-containing functional groups, which can enhance their water solubility, surface reactivity, and functionalization potential. These properties expand the application fields of carbon dots, helping to reduce carbon emissions and achieve sustainable development goals. In this review, we detail the synthesis methods of B-CDs and elucidate the formation mechanisms of carbon dots derived from three major biomass carbon sources, cellulose, starch, and chitosan, focusing on their structures and carbonization processes. Furthermore, we systematically interpret the four primary photoluminescence (PL) mechanisms of B-CDs: quantum confinement effect, surface state emission, molecular state emission, and carbon core photoluminescence. The fluorescence emission of B-CDs results from the combined action of these mechanisms. The structural characterization of B-CDs is then introduced. Key PL properties, including tunable fluorescence emission, fluorescence quenching phenomena, and quantum yield, are comprehensively outlined. The latest research progress of B-CDs in sensor, anti-counterfeiting, bioimaging, and photoelectric fields is summarized. In each category, representative cases are carefully selected to elaborate the underlying working mechanism of biomass carbon dots in application fields, aiming to explore the application ideas and design concepts of biomass carbon dots.

Although significant progress has been made in the study of B-CDs, the reproducibility and batch consistency of their synthesis remain to be solved. Since natural biomass often contains polysaccharides, proteins, lipids and other components, the complexity of its precursors will lead to significant differences in the size distribution, surface functional group composition, and emission center concentration of carbon dots, which will affect the quantum yield and optical stability. Therefore, future research should systematically evaluate the pyrolysis path and carbonization efficiency of different carbon sources (such as cellulose, starch, chitosan) under pyrolysis or hydrothermal conditions and establish a strict process route against parameters such as reaction temperature, incubation time, and reaction system pH to improve the repeatability of synthesis. In addition, carbon sources, dopants and preparation methods play a key role in the formation of B-CD, but their effects on the formation process and mechanism of B-CD need further study. Different biomass carbon sources have significant differences in structure and chemical composition. These differences determine the variations in the types and reactivity of intermediate products during hydrothermal or microwave-assisted carbonization processes. Dopants (N, S, P, B, etc.) are also crucial in regulating the electronic structure and surface state of carbon dots. By controlling the dopant/carbon source molar ratio and precursor solvent environment, intermediate energy levels and surface functional group distribution can be accurately adjusted. Moreover, the preparation of B-CDs is still largely in the laboratory stage, and large-scale industrial production is crucial for functional applications of B-CDs. Therefore, future research should focus on how to precisely regulate the synthesis of B-CDs based on the varied formation mechanisms and how to achieve large-scale industrial preparation of biomass carbon dots with high quantum yield. The purpose of this study is to provide inspiration for future research and the utilization of biomass carbon point to promote the theoretical research and industrial production application of biomass carbon point.

## Figures and Tables

**Table 1:** Overview of top-down synthesis of carbon dots.

Synthesis Method	Topic	Advantages	Disadvantages
Arc-Discharge Method	High-temperature (50–200 A) and high-pressure (0.05–40 kPa) arc evaporation decomposition.	Simple process; diverse carbon sources; large scale.	Complex process; poor size control; low yield.
Laser Ablation Method	Laser beam focusing resolution.	Fast; effective; highly adjustable.	Low yield; poor size control; high equipment requirements.
Chemical Etching Method	Strong acid carbonizes small organic molecules.	Simple process.	Complex process; harsh conditions, many steps; poor control over size.

**Table 2:** Overview of bottom-up synthesis of carbon dots.

Synthesis Method	Topic	Advantages	Disadvantages
Hydrothermal Synthesis	Reaction with solvent in high temperature (180–280 °C) and high pressure (0.1–4.0 MPa) water environment.	Environmentally friendly; easy operation; mild conditions.	Longer reaction time; harsh conditions.
Microwave Synthesis	Microwave radiation heating (150–300 °C).	Fast reaction; high yield; enables rapid synthesis.	High equipment requirements; poor size control.
Pyrolysis Synthesis	High-temperature (200–900 °C) pyrolytic carbonization of organic precursors.	Simple procedure; high yield.	High energy cost.

**Table 3:** Comparative analysis of different biomass precursors.

Carbon Source	Synthesis Method	Heteroatom Dopant(s)	Carbon Dot C Content (at%)	Scalability	Reference
Rice straw	One-step hydrothermal	N: ethylenediamine, S: lipoic acid	67.71	Low	[25]
Dialdehyde starch	One-step hydrothermal	N: 1,8-diaminonaphthalene	67.86	Low	[26]
Chitosan	One-step hydrothermal	N: ethylenediamine	Not reported	Low	[27]
Glucose	One-step hydrothermal	N: Urea	Not reported	Low	[28]
Grapefruit juice	One-step hydrothermal	N: Urea	58.91	Low	[29]
Citric acid	One-step hydrothermal	N: Urea	Not reported	Low	[30]
Carboxymethyl cellulose	One-step hydrothermal	N: Urea	Not reported	Low	[31]

**Table 4:** Statistics of QY for B-CDs reported in the literature.

Raw Material	Solvent	Dopant	Ex/Em (nm)	Meas. Temp.	QY	Reference
Rice straw	Water	N: Ethylenediamine; S: Lipoic acid	350/440	Room temperature	62.8%	[25]
Glucose	Water	N: Urea	340/435	Room temperature	14.9%	[28]
o-Phenylenediamine	Water	N: o-Phenylenediamine; F: 2,3,5,6-Tetrafluoroterephthalic acid	428/540	Room temperature	52.2%	[58]
o-Phenylenediamine	Water	P: Ammonium dihydrogen phosphate; Br: KBr	450/610	Room temperature	11.3%	[59]
Grapefruit juice	Water	N: Urea	430/512	Room temperature	84.9%	[29]
Methyl cellulose	Water	N: Ethylenediamine; S: L-Cysteine	330/370	Room temperature	12.3%	[60]
Citric acid	Water	N: Urea	360/435	Room temperature	26.6%	[30]
Carboxymethyl cellulose	Water	N: Urea	350/470	Room temperature	35.5%	[31]
Citric acid	Water	N: Triethylenetetramine	360/440	Room temperature	70%	[61]
Spinach	Ethanol/Acetone	/	365/493	Room temperature	10.8%	[51]
Calcein	Ethanol/NaOH	/	490/530	Room temperature	21.9%	[52]
